# Jumping into a Healthier Future: Trampolining for Increasing Physical Activity in Children

**DOI:** 10.1186/s40798-021-00335-5

**Published:** 2021-07-30

**Authors:** Isabelle Schöffl, Benedikt Ehrlich, Kathrin Rottermann, Annika Weigelt, Sven Dittrich, Volker Schöffl

**Affiliations:** 1 Department of Pediatric Cardiology, University Hospital Erlangen-Nuremberg, 91054 Erlangen, Germany; 2 School of Clinical and Applied Sciences, Leeds Beckett University, Leeds, Great Britain; 3 Section of Sportsmedicine and Sports Orthopaedics, Department of Orthopedic and Trauma Surgery, Klinikum Bamberg, Bamberg, Germany; 4 Section of Wilderness Medicine, Department of Emergency Medicine, University of Colorado School of Medicine, Denver, USA

**Keywords:** Cardiopulmonary function, Exercise testing, Rehabilitation, Training modalities, Pediatric cardiology, Training, Children, Pediatrics, Physical activity

## Abstract

**Objectives:**

Physical activity in children and adolescents has positive effects on cardiopulmonary function in this age group as well as later in life. As poor cardiopulmonary function is associated with higher mortality and morbidity, increasing physical activity especially in children needs to become a priority. Trampoline jumping is widely appreciated in children. The objective was to investigate its use as a possible training modality.

**Methods:**

Fifteen healthy children (10 boys and 5 girls) with a mean age of 8.8 years undertook one outdoor incremental running test using a mobile cardiopulmonary exercise testing unit. After a rest period of at least 2 weeks, a trampoline test using the mobile unit was realized by all participants consisting of a 5-min interval of moderate-intensity jumping and two high-intensity intervals with vigorous jumping for 2 min, interspersed with 1-min rests.

**Results:**

During the interval of moderate intensity, the children achieved $\dot{V}{O}_2$-values slightly higher than the first ventilatory threshold (VT1) and during the high-intensity interval comparable to the second ventilatory threshold (VT2) of the outdoor incremental running test. They were able to maintain these values for the duration of the respective intervals. The maximum values recorded during the trampoline test were significantly higher than during the outdoor incremental running test.

**Conclusion:**

Trampoline jumping is an adequate tool for implementing high-intensity interval training as well as moderate-intensity continuous training in children. As it is a readily available training device and is greatly enjoyed in this age group, it could be implemented in exercise interventions.

## Key Points

- Trampolining is a widely used physical activity in children and adolescents.
- As the proportion of children achieving the required 60 min of physical daily activity has declined dramatically over the last years, motivating children and developing adequate training methods are crucial.
- High-intensity interval training (HIIT) provides a more time-efficient approach with a higher fun aspect, underlining the need for developing age-appropriate training methods with a focus on HIIT.
- Trampolining achieves intensities of oxygen uptake ($\dot{V}{O}_2$) and heart rate above the second ventilatory threshold and is therefore suitable for HIIT.
- This is the first study using cardiopulmonary exercise equipment for evaluating trampolining for its use for HIIT in children.

## Introduction

Cardiorespiratory fitness (CRF) is an objective, reproducible, and physiological measure [1] that can be recorded by using maximal graded cardiorespiratory exercise testing and determining peak oxygen uptake ($\dot{V}{O}_{2 peak}$) [2].

Evidence of the association between low CRF and a higher morbidity and mortality from all causes, including cardiovascular disease and cancer, has been consistent and strong [3]. An improvement of CRF by 1 ml/kg/min (determined using a maximal bike test) has been shown to reduce the risk for developing overweight or obesity by 10% in 6 years [4]. In addition to that, improving CRF in childhood and adolescence is associated with a healthier cardiovascular profile in later life [5]. Furthermore, a physically active childhood enhances a physically active lifestyle over a life span [6].

As CRF is an objective reproducible physiological measure reflecting the functional influences of physical activity habits, genetics, and disease status [7], the aim should be to improve CRF early in life. However, currently CRF of 25.4 million people aged 6 to 19 years from 27 countries has declined by 3.6% per decade from 1985 to 2003 [8]. Even though the WHO recommends that children spend at least 60 min/day of moderate-to-vigorous-intensity physical activity [9], most children spend an average of 8 to 9 h/day in sedentary behavior which in turn tends to increase by 30 min/day every year in school-aged children [10]. Consequently, the number of children and adolescents with low CRF gradually increases [11]. It is therefore essential to devise effective exercise modalities fit for the use in very young children to change their general movement pattern towards a higher level of physical activity.

There are two training modalities which have been explored in the literature: moderate-intensity continuous training (MICT) and high-intensity interval training (HIIT). In HIIT, short bursts of high-intensity activity alternate with lower-intensity activity for recovery or rest [12–14]. The intensity for the high-intensive bursts is described as “all-out,” “maximal effort,” “≥ 90% VO_2_peak,” “85–95% maximum heart rate (HR),” or “≥ 100% maximal aerobic speed” [14]. MICT on the other hand consists mainly of aerobic exercise training performed continuously or in intervals [15]. The training modality which shows the most promising effects for improving CRF in children, adolescents, and adults is high-intensity interval training (HIIT) [14, 16]. Possible explanations for the success of HIIT compared to MICT are the differing adaptations induced in the mitochondria in the trained muscles and a higher effect on central adaptation such as maximal stroke volume, cardiac output, and blood volume, all being important components of CRF [17–19]. Maybe even more important than its effects is the smaller time commitment which could increase training adherence [20]. Intermittent exercise also represents children’s spontaneous physical activity [21] and may therefore be better received in this age group than MICT.

Since HIIT seems to represent an effective approach to achieving improvements in CRF among healthy children and adolescents, we aimed to evaluate a training modality which could be easily accessed by healthy children and already represents an activity widely recognized in that age group: trampolining. A first approach to measuring the intensity of trampoline jumping in children has involved the measuring of accelerometry (ACC)-derived and muscle electromyography (EMG)-based estimates [22]. However, the two measurements provided different results, with ACC describing trampoline jumping as being of vigorous intensity as a consequence of the body being hurled through space [22]. But EMG categorized it as being of light and moderate intensity as it only involves brief bursts of muscle activity [22]. Two studies have investigated the physiological demands of trampolining in adults and concluded that trampolining achieved comparable $\dot{V}{O}_2$-values as treadmill running at the same intensity [23, 24]. The conclusion of the authors was that even though the cardiopulmonary parameters were significantly higher during trampolining compared to treadmill running, the fact that the trendline associated with each variable was similar, monitoring the intensity of the session based on heart rate during trampolining as is already the custom with running was justified [24].

The question remains, whether trampoline jumping can be categorized as a vigorous or as a moderate-intensity physical activity and whether it could be used as a reasonable training modality for HIIT in children.

## Material and Methods

The study was performed in accordance with the standards of ethics outlined in the Declaration of Helsinki and was approved by the Ethics Committee of the University of Erlangen-Nürnberg, FRG (No. 409_19).

### Subjects

Fifteen healthy children aged between 7 and 10 years agreed to participate in this study. Each child as well as their legal guardians gave written informed consent using a consent form approved by the Ethics Committee of the University of Erlangen-Nuremberg, FRG. All participants were Caucasian, non-obese, and healthy and were recruited from local schools. All children performed one outdoor incremental running test and then one trampoline jumping test during which the children were asked to jump at different intensities according to a preset protocol.

### Body Composition

Height and weight were measured using a stadiometer and electronic scale (Seca 704 S, Hamburg, Germany).

### Measurement of $\dot{V}{O}_2$

The cardiopulmonary exercise test was performed using a mobile testing device (Metamax ®, Cortex, Leipzig, Germany). A small, low-dead-space respiratory valve (88 ml) with a pediatric mouthpiece and headgear was used. Gas-exchange was measured continuously during each test using a breath-by-breath method and averaged over 15-s intervals. Criteria for completion of a valid peak $\dot{V}{O}_2$ test included two of the following three criteria: (1) HR ≥ 200 beats/min (bpm), (2) respiratory exchange ratio (RER) ≥ 1.0, and (3) volitional fatigue [25]. All children were instructed to abstain from food or carbohydrate-rich drinks for 2 h leading up to the test. Ventilatory thresholds VT_1_ and VT_2_ were determined using the V-slope method proposed by Wasserman et al. [26].

### Outdoor Incremental Running Test Protocol

The test was performed outdoors using an incremental step test previously described [27]. After a warm-up and a short rest period, each child was equipped with a heart rate monitor (Polar H7 Bluetooth Smart 4.0 ® heartrate sensor, Kempele, Finnland) and the mobile exercise equipment. The mask was fitted and the backpack containing the mobile exercise equipment (MetaMax 3B®, Cortex, Leipzig, Germany) adjusted, so it would not move on the child’s torso.

The test consisted of 4 steps of 2-min lengths. The first 2 min consisted of walking at a leisurely pace. After that, the children were instructed to increase their speed to an easy jog. After 2 min at this speed, they were instructed to run with some effort for another 2 min and then to increase their speed to as fast as possible and maintain this speed for as long as possible. Once they could no longer maintain their maximum speed or were too exerted to keep going, the test was ended. An experienced researcher and running coach for children performed the whole test alongside each child controlling the speed with a GPS sensor (Garmin Fenix 5S®, Garmin, Olathe, USA). This was important for slowing the children down during the first two steps and then to motivate them during the last step. Running was performed on a flat and even trail and children were instructed to use sportive footwear and clothing. We did not perform a treadmill test, as this outdoor incremental running test protocol has proven its efficacy this age group [28].

### Trampoline Test Protocol

A minimum of 2 weeks later, all children were tested again on the trampoline. They were first fitted with the mobile cardiopulmonary exercise testing device and the heart rate monitor as described above. To assess the intensity during leisurely jumping as well as during vigorous exercise, the protocol consisted of 5 min of easy hopping. During these first 5 min, the children were instructed to jump very little and to not overexert themselves. After this phase of moderate intensity, followed a rest period of 1 min which the children spent standing still. Then, followed 2 min of vigorous jumping, during which the children were motivated to jump as high as possible. After a second rest period of 1 min spent standing still another bout of vigorous exercise was performed for another 2 min. All recorded values were then averaged over the duration of each interval. Additionally, the maximum values reached by each child during the periods of vigorous jumping were recorded.

### Statistical Analysis

Statistical analysis was performed using Microsoft Excel 2000® for data collection and SPSS 12.0® (SPSS Inc., Chicago, IL). All measured values are reported as means and standard deviations. The Kolmogorov-Smirnov test was used to check for normal distribution. Homogeneity of variance was investigated using Levine’s F-test. For normally distributed variables, differences between the two test protocols were assessed with paired t-tests; otherwise, the Wilcoxon or the Whitney-Mann U-tests were used. All tests were 2-tailed; a 5% probability level was considered significant (*).

## Results

### Subjects

We were able to test 15 children outdoors and on the trampoline. Out of the 15 children (mean age 8.8 years, mean weight 27.6 kg, height 133.4 cm, body mass index (BMI) 15.3 kg^−2^), 10 were boys (mean age 8.8 years, weight 28.9 kg, height 135.7 cm, BMI 15.5 kg^−2^) and 5 were girls (mean age 8.7 years, weight 25 kg, height 128.8 cm, BMI 15 kg^−2^). The boys and girls did not differ significantly from each other with respect to age or anthropometric variables. When asked about the subjective effort, all children felt tired after having performed the trampoline test but also agreed that they had enjoyed themselves.

### Oxygen Uptake ($\dot{V}{O}_2\Big)$

**Fig. 1:**
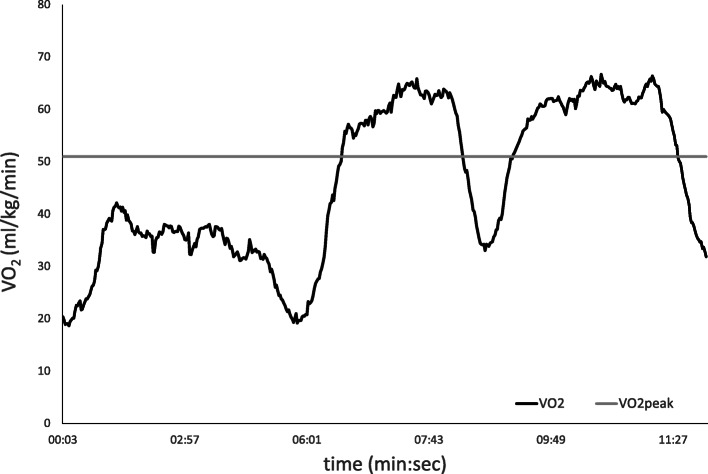
An example of $\dot{\boldsymbol{V}}{\boldsymbol{O}}_{\mathbf{2}}$ over the course of the trampoline test along with the $\dot{\boldsymbol{V}}{\boldsymbol{O}}_{\mathbf{2}}$-peak value recorded for the outdoor incremental running test of one child (VO_**2**_, oxygen uptake)

**Fig. 2:**
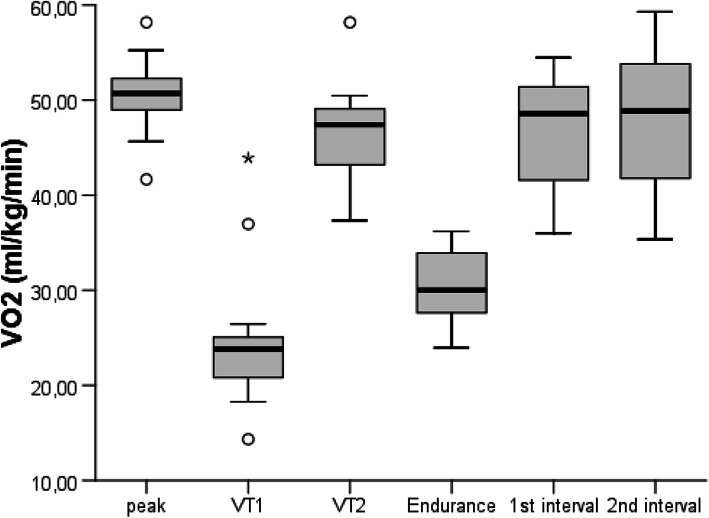
Median values of the oxygen uptake ($\dot{\boldsymbol{V}}{\boldsymbol{O}}_{\mathbf{2}}$) at the first (VT1) and second (VT2) ventilatory threshold, and the median values recorded for the interval of moderate intensity, the first and second high-intensity intervals as well as the maximum oxygen uptake during the trampoline test, alongside with the values for minimum, maximum, and the 25th and 75th quartile of all participants (degree symbol denotes far outliers (1 interquartile range of 1.5) and asterisk denotes extreme outliers (2 interquartile ranges of 1.5))

An example of $\dot{V}{O}_2$ over the course of the trampoline test is represented in Fig. 1 along with the $\dot{V}{O}_2$-peak value recorded for the outdoor incremental running test for this child. The oxygen uptake values of all the participants are represented in Fig. 2 as well as in Table 1. The mean $\dot{V}{O}_2$ achieved during the 5 min of trampoline jumping of moderate intensity was significantly higher than the $\dot{V}{O}_2$ at VT1 determined during the outdoor incremental running test. During both intervals of vigorous jumping, comparable values to VT2, determined during the outdoor incremental running test, were achieved. When compared to $\dot{V}{O}_{2 peak}$ from the outdoor incremental running test, the mean values during the first interval of higher intensity were significantly lower but were comparable during the second 2-min interval of higher intensity. The maximum $\dot{V}{O}_2$ determined during the trampoline jumping was significantly higher than $\dot{V}{O}_{2 peak}$ determined during the outdoor incremental running test.

**Table 1:** Mean values recorded during the incremental running outdoor test compared to the mean values for the three jumping intervals as well as the mean values of the maximum achieved by each child during the trampoline jumping test ($\dot{\boldsymbol{V}}{\boldsymbol{O}}_{\mathbf{2}}$ oxygen uptake, *O*_*2*_*-pulse* oxygen pulse, $\dot{\boldsymbol{V}}\boldsymbol{E}$ minute ventilation, *VT1* first aerobic threshold, *VT2* second ventilatory threshold)

	Running test			Trampoline test			Maximum
	Peak	VT1	VT2	Endurance	1st interval	2nd interval	
$\dot{\boldsymbol{V}}{\boldsymbol{O}}_{\mathbf{2}}$ **(mL kg**^**−1**^ **min**^**−1**^**)**	50.4 (4)	24.6 (7.3)	46.2 (5.4)	30.4 (4.1)	47.1 (5.6)	48.3 (6.9)	55.6 (6.7)
**O**_**2**_**-pulse (mL beat**^**−1**^**)**	7.4 (1.9)			6.2 (1.1)	7.4 (1.6)	7.5 (1.8)	8.2 (2)
**Heart rate (min**^**−1**^**)**	191.7 (8.3)	130.4 (12.6)	183 (10.3)	133.6 (10.8)	173.8 (10.2)	183 (10.3)	189.6 (8.3)
$\dot{\boldsymbol{V}}\boldsymbol{E}$ **(L min**^**−1)**^	53.9 (11.2)			24.3 (3)	45.6 (9.3)	47 (11)	52.9 (12)

### Heart Rate

The heart rate values for the trampoline and the outdoor incremental running test are represented in Table 1. The mean heart rate during the interval of moderate intensity was comparable to the heart rate at VT1 during the outdoor incremental running test. The same was true for the heart rate at VT2 and the HR during the second high-intensity interval which were also comparable. The maximum heart rate achieved during the trampoline test was not significantly higher than the one achieved during the outdoor incremental running test.

### Oxygen Pulse (O_2_-pulse) and Minute Ventilation ($\dot{V}E$)

The parameters for O_2_-pulse and$\dot{V}E$ are represented in Table 1. The peak O_2_-pulse was comparable to the O_2_-pulse during both high-intensity intervals and significantly higher than during the interval of moderate intensity. The maximum O_2_-pulse recorded during the trampoline test was significantly higher than during the outdoor incremental running test. Mean peak $\dot{V}E$ was significantly higher during the outdoor incremental running test than during the trampoline test even though the maximum recorded value of $\dot{V}E$ during the trampoline test was comparable to peak $\dot{V}E$ during the outdoor incremental running test. Accordingly, the mean $\dot{V}E$ recorded during the interval of moderate intensity was also significantly lower than the peak $\dot{V}E$ from the outdoor incremental running test.

## Discussion

Trampoline jumping is a widely used physical activity in children. Therefore, trampoline jumping represents an ideal means for promoting physical activity in children. In order to evaluate its intensity during different jumping modalities, we investigated slow jumping with moderate intensity over a period of 5 min and high-intensity jumping over a period of 2 min.

### Eligibility of Trampoline Jumping for MICT and HIIT

The first interval of the trampoline test which consisted of slow and mild jumping was supposed to represent a moderate continuous intensity. However, $\dot{V}{O}_{2 peak}$ during this first interval was already higher than $\dot{V}{O}_2$ at VT1 from the outdoor incremental running test. Still, the heart rate achieved during this interval of moderate jumping and the HR achieved at VT1 during the outdoor incremental running test was comparable, indicating that the heart rate is not an accurate tool for estimating cardiopulmonary exertion. The same observation was made for $\dot{V}{O}_2$ at VT2 during the outdoor incremental running test which was comparable to $\dot{V}{O}_2$ during both high-intensity intervals. However, the mean heart rate was significantly lower during the first high-intensity interval on the trampoline when compared to the VT2 of the outdoor incremental running test. The mean heart rate on the trampoline only rose enough during the second high-intensity interval on the trampoline to reach comparable values to the HR recorded at VT2 during the outdoor incremental running test. Slow trampoline jumping could therefore be an adequate tool for moderate-intensity continuous training in children as it can be performed close to $\dot{V}{O}_2$ at VT1. However, the children stated that they were bored during the slow jumping and felt no tiredness afterwards. The vigorous jumping was much more enjoyable according to their accounts and was performed with $\dot{V}{O}_2$-values comparable to VT2 from the outdoor incremental running test, thus representing high-intensity interval training.

The mean $\dot{V}{O}_2$ achieved during the second high-intensity interval was comparable to $\dot{V}{O}_{2 peak}$ from the incremental step test, and the maximum $\dot{V}{O}_2$ recorded during the trampoline test was significantly higher. Additionally, the children were not only able to achieve this high-oxygen uptake during vigorous trampoline jumping but were also able to sustain this high intensity for a duration of 2 min as can be seen in the exemplary data represented in Fig. 1. These findings suggest that a true $\dot{V}{O}_{2\mathit{\max}}$ can be achieved during trampoline jumping by kids.

Two previous studies have investigated the cardiopulmonary effects of trampolining [23, 24]. The maximum $\dot{V}{O}_2$ recorded during vigorous jumping was around 40 ml kg^−1^ min^−1^ in healthy, adult males [23, 24], which is significantly lower than the maximum recorded in our study (55.6 ml kg^−1^ min^−1^). Children tend to achieve higher $\dot{V}{O}_2$-values than adults during cardiopulmonary exercise tests [29], which can explain this discrepancy. In these studies, $\dot{V}{O}_2$-values during vigorous trampolining did not differ significantly from vigorous running on the treadmill [23, 24], whereas the children in our study achieved significantly higher values on the trampoline than during the outdoor incremental running test. One explanation could be that the children were maybe more willing to fully exhaust themselves on the trampoline than while running outdoors. It could also be that larger muscle groups are recruited during vigorous trampolining than when running. Comparable test protocols are needed to evaluate this finding further.

### Cardiac and Pulmonary Contribution During Trampoline Jumping

The cardiac output, indirectly measured through the O_2_-pulse, was comparable during both high-intensity intervals and at $\dot{V}{O}_{2 peak}$ during the outdoor incremental running test. Interestingly, the minute ventilation $\dot{V}E$ was significantly lower during both high-intensity intervals than at $\dot{V}{O}_{2 peak}$ during the outdoor incremental running test, but the maximum value during trampoline jumping and incremental step test was comparable. This indicates that peak O_2_-pulse was reached by the children during the trampoline test and that they were able to maintain this high cardiac output for the full duration of the high-intensity interval. On the other hand, even though peak $\dot{V}E$ reached during trampoline jumping and outdoor running was comparable, the mean value for $\dot{V}E$ recorded during the high-intensity interval on the trampoline was lower than peak $\dot{V}E$ recorded during the outdoor incremental running test. This could be due to a slow rise of $\dot{V}E$, reaching maximum values only towards the end of the 2-min interval. Since mean O_2_-pulse and mean HR achieved during the vigorous trampoline jumping were comparable to peak O_2_-pulse and peak HR from the outdoor incremental running test, the significant difference between mean $\dot{V}E$ achieved during the trampoline test and peak $\dot{V}E$ achieved during the outdoor incremental running test underlines the fact that the main limitation during maximum exertion is of a cardiac and not a pulmonary nature. The children reach their peak O_2_-pulse and heart rate so early in the 2-min interval of vigorous trampolining as to reach mean values that are comparable to peak O_2_-pulse and HR recorded during the outdoor incremental running test. However, they only reached the peak $\dot{V}E$ from the outdoor incremental running test at the very end of this 2-min interval of vigorous jumping.

This study has several limitations. First, the number of participants is small and more significant differences might have become apparent with a higher number of children. There was no evaluation of the power realized during the trampoline jumping as the determination of metabolic equivalents for trampoline jumping has not been established yet.

## Conclusion

As the test subjects in this study were able to achieve $\dot{V}{O}_2$-values at VT2 which they were then able to maintain for a duration of 2 min twice with only 1 min rest in between, trampoline jumping could be a very effective way to achieve HIIT in young children. The children all stated that they had tremendously enjoyed the vigorous jumping and more than the slow jumping of moderate intensity. As it is a readily available training device for most families—either at home or in a near-by facility—it could be used for promoting physical activity in children through high-intensity interval training interventions.

## Data Availability

Can be made available upon reasonable request

## Abbreviations

VT1: First ventilatory threshold
VT2: Second ventilatory threshold
HIIT: High-intensity interval training
$$\dot{V}{O}_2$$: Oxygen uptake
$\dot{V}{O}_{2 peak}$: Peak oxygen uptake
CRF: Cardiorespiratory fitness
MICT: Moderate-intensity continuous training
HR: Heart rate
ACC: Accelerometry
EMG: Electromyography
RER: Respiratory exchange ratio
BMI: Body mass index
O_2_-pulse: Oxygen pulse
$$\dot{V}E$$: Minute ventilation
